# Periarticular Injection Versus Peripheral Nerve Blockade in Bilateral Total Hip Arthroplasty

**DOI:** 10.7759/cureus.39503

**Published:** 2023-05-25

**Authors:** Carolyn Sivco, Dahlia Townsend, Michael P Leslie, Jinlei Li

**Affiliations:** 1 Anesthesiology, Yale School of Medicine, New Haven, USA; 2 Anesthesiology, University of Pittsburgh Medical Center, Pittsburgh, USA; 3 Orthopedics and Rehabilitation, Yale School of Medicine, New Haven, USA

**Keywords:** bupivicaine, femoral nerve block, lateral femoral cutaneous nerve block, quadratus lumborum block, post operative pain management, perioperative pain management, periarticular injection, peripheral nerve blockade, total hip arthroplasty (tha)

## Abstract

Pain control after total hip arthroplasty is associated with patient satisfaction, early discharge, and improved surgical outcomes. Two commonly utilized opioid-reducing analgesic modalities are periarticular injection (PAI) by surgeons and motor-sparing peripheral nerve block (PNB) by anesthesiologists. We present a case contrasting PAI and PNB in a single patient undergoing bilateral total hip arthroplasty. For the left hip, the patient received preoperative transmuscular quadratus lumborum, femoral nerve, and lateral femoral cutaneous nerve blocks using a combination of low-concentration local anesthetic and glucocorticoids. For the right hip, the patient received an intraoperative PAI with liposomal bupivacaine. The patient's pain scores and recovery were evaluated for three months postoperatively. The patient's pain scores on postoperative day (POD) zero to five were consistently lower in the left hip than in the right hip. For this patient undergoing bilateral hip replacement, preoperative PNBs were superior to PAI for postoperative pain control.

## Introduction

Current pain control modalities following total hip arthroplasty include systemic analgesia (with and without opioids), neuraxial analgesia, peripheral nerve blocks (PNBs), and periarticular injection (PAI) [1]. PNBs are of unique interest as they have the potential to improve not only immediate postoperative pain but also long-term outcomes for patients, including lower risk of chronic post-surgical pain and improved function. In this case, we sought to determine if there was a difference in postoperative pain scores and recovery for this patient receiving PNB versus PAI.

## Case presentation

**Figure 1:**
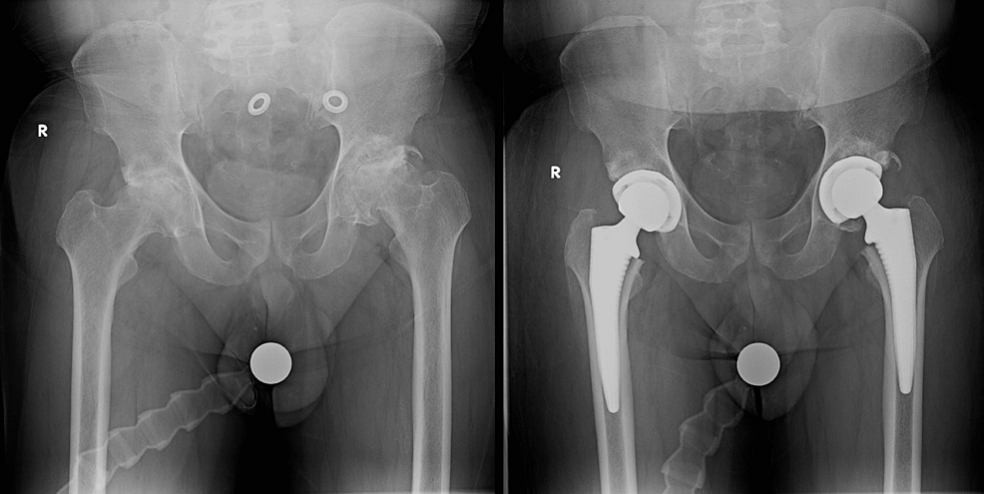
Preoperative (left) and postoperative (right) X-rays of bilateral hip joints

A 5'4" (1.626m), 69.4kg, American Society of Anesthesiologists (ASA) 2 male in his 50s presented for elective bilateral total hip arthroplasty due to advanced osteoarthritis. His medical history was otherwise unremarkable. His preoperative worst pain scores were 7 out of 10 in the right hip and 8 out of 10 in the left hip. His pain had become increasingly debilitating, especially with decreased physical activity in the setting of the Covid-19 pandemic. He was able to walk up to two miles per day, but reported difficulty with ambulation requiring an assistive device at times. The patient's preoperative and postoperative X-rays can be found in Figure 1.

On the day of surgery, the patient was consented for left-sided preoperative PNBs and right-sided intraoperative PAI with the understanding that we wanted to compare these two analgesic modalities for postoperative pain relief. The PNBs were performed under direct ultrasound guidance (X-Porte C60, SonoSite, Bothell, Washington) by the anesthesiologist. The patient received sedation with 2mg midazolam and 100mcg fentanyl intravenously. The patient was placed in the right lateral decubitus position. A transmuscular quadratus lumborum (QL) block was first performed using a 20-gauge insulated, short-bevel nerve block needle with a total of 30mL of 0.2% ropivacaine, 40mg methylprednisolone acetate (MPA), and 5mg dexamethasone sodium phosphate (DEX). The patient was then turned supine, and both a femoral nerve block and lateral femoral cutaneous nerve (LFCN) block were performed using 10mL of 0.2% ropivacaine, 20mg MPA, and 2.5mg DEX in each block. The needle, nerve or plane, and local anesthetic spread were easily visualized at all times under ultrasound guidance, and there were no immediate complications, including aspiration of blood, pain, resistance on injection, or paresthesia.

In the operating room, the patient received an additional 2mg of midazolam and 100mcg fentanyl for sedation. An L3-L4 combined spinal/epidural was performed intraoperatively for the primary anesthetic and for opioid-sparing analgesia. A total of 3mL of 0.5% bupivacaine was administered intrathecally, followed by placement of an epidural catheter. A total of 13 mL of 0.25% bupivacaine was administered epidurally throughout the procedure. The procedure was performed bilaterally via the direct anterior approach on a Hana table (OSI, Medine, Minnesota). The total surgical time was 152 minutes. At the end of the procedure, the surgeon performed a PAI using liposomal bupivacaine mixed with 30 ml 0.5% plain bupivacaine on the right hip. The local injection was performed with a 20-gauge needle into the closed capsule of the hip joint, the reflected head of the rectus, the tensor fascia lata, and the subcutaneous tissues. The estimated total blood loss for the procedure was 1200mL, and the patient received 409mL of autologous transfusion via the CellSaver. There were no complications, and the patient was transferred to the post-anesthesia care unit (PACU) in stable condition.

In the PACU immediately following surgery, the patient denied any pain. An epidural infusion of 0.1% bupivacaine at 8mL per hour was started at 4pm, approximately two hours after his arrival in the PACU. The patient continued to deny pain and was transferred to his hospital room at 6pm. Later that night, around 11pm, the patient had increasing pain which improved with 10mg oxycodone. On postoperative day (POD) one, the patient reported right hip pain greater than left hip pain, controlled with scheduled acetaminophen (975mg orally every 6 hours), scheduled ketorolac (15mg intravenously every six hours), and 5-10mg oxycodone as needed (total 25mg). On exam, he had swelling to bilateral incision sites, right greater than left. Motor function of his bilateral lower extremities was intact except for reduced strength (4/5) with right hip flexion. Sensation to his bilateral lower extremities was intact except for numbness to the bilateral upper thighs in the setting of the epidural. The epidural infusion was discontinued, and the catheter was removed on POD one. The patient was able to ambulate with physical therapy multiple times throughout POD one with minimal assistance.

On POD two, the patient continued to have right hip pain greater than left hip pain. He continued to work with physical therapy with minimal assistance. He reported residual numbness to the bilateral hips and buttocks. He was discharged home later that afternoon. The patient reported taking 5mg oxycodone at home later that evening as a preventative measure rather than for ongoing pain. On POD three, the patient continued to take oxycodone as a preventative measure, as well as acetaminophen (975mg three times a day) and meloxicam (15mg daily). He reported minimal pain, right greater than left. He participated in daily home physical therapy. On PODs four to five, the patient reported minimal pain, right greater than left. He reported greater swelling to the right hip causing "discomfort with movement, but not much pain".

After POD five, the patient denied any pain at rest. The swelling to his left hip resolved on POD 13, while the swelling to his right hip resolved on POD 34. He transitioned to outpatient physical therapy on POD 20 and continued weekly physical therapy sessions until POD 69. By POD 51, the patient resumed his regular exercise routine, including running and playing basketball with his grandson. A summary of the patient's daily postoperative pain scores, postoperative opioid consumption, and postoperative pain comparison between the left and right hips can be found in Tables 1 and 2.

**Table 1:** The patient's average daily pain scores at rest and postoperative opioid consumption POD - postoperative day; MME - milligram morphine equivalents

Time	Left hip pain score	Right hip pain score	Total mg oxycodone	Oral MME
Pre-op	8	7	0	0
POD 0	0	0	10	15
POD 1	6	8	25	37.5
POD 2	3	4	35	52.5
POD 3	0	1	25	30
POD 4	0	1	0	0
POD 5	0	1	0	0
POD 6-69	0	0	0	0

**Table 2:** The patient’s subjective pain comparison between the left and right hip over time PACU - post-anesthesia care unit; POD - postoperative day; LPNB - left hip that received peripheral nerve blocks; RPAI - right hip that received peri-articular injection. * More swelling noted at right hip, causing limited range of motion. ** Bilateral swelling resolved.

Timeline	Pain comparison
Pre-op	L_PNB > _R_PAI_
PACU/POD 0	L_PNB = _R_PAI_
POD 1-5	L_PNB < _R_PAI_
POD 6-34	L_PNB = _R_PAI *_
POD 35-69	L_PNB =_ R_PAI **_

## Discussion

This case report is unique because, preoperatively, this patient's bilateral hip pain and the morphology of his degenerative disease were both radiographically and clinically similar in duration and disability. This is often a confounding factor in comparing perioperative analgesia between patients with similar diagnoses but dissimilar severity. As a result, we were able to contrast two very common analgesia modalities for hip replacement surgery in a single patient. Ultimately, the patient consistently reported better postoperative pain control on the left hip with PNB than on the right hip with PAI.

The transmuscular QL, femoral, and LFCN blocks were chosen for this patient for a variety of reasons. The transmuscular QL block has been shown to provide effective analgesia and reduce opioid consumption as compared to no PNB or PAI following total hip arthroplasty (THA) [2]. In addition, the transmuscular QL block has been shown to provide equivalent pain reduction when compared to the lumbar plexus nerve block following THA but with fewer side effects, such as lower extremity weakness [3]. The femoral nerve block can cause quadriceps weakness but has also been shown to reduce time to first analgesic request, time spent in PACU, and overall opioid consumption during the first 24 hours after hip surgery [4]. To minimize weakness and maximize pain control, we used low concentration local anesthetic. The LFCN block is a purely sensory blockade with no risk of lower extremity weakness and a large coverage area extending from the knee to the posterolateral gluteal region [5]. The LFCN block can be helpful for postoperative pain management, though its efficacy when used alone in THA is controversial [4]. We chose the combination of transmuscular QL, femoral, and LFCN blocks to minimize the risk of lower extremity weakness and for the documented efficacy in dermatomal coverage, with the transmuscular QL block covering from T6-7 to L1-2, the femoral nerve block covering L2-4, and the LFCN block covering L2-3.

Generally speaking, single administration PNB or PAI without adjuvants seldom lasts up to 24 hours; therefore, various local anesthetic adjuvants have been adopted by practitioners. DEX and MPA are among the commonly utilized medications in PNBs and neuraxial nerve blocks, respectively, to augment the block effect and prolong the duration of analgesia. In addition, MPA is used widely in various chronic pain procedures and has demonstrated efficacy for long-term post-surgical pain control [6]. DEX is water-soluble, has a faster onset than MPA, and prolongs the duration of local anesthetics for about six to eight hours on average [7]. On the other hand, MPA has variable onset that may take up to 24 hours. Therefore, MPA alone has been used extensively in chronic pain management but not in acute pain control. For these reasons, in this case, DEX was utilized as a "bridge" between the local anesthetic and the longer-acting MPA [8].

There are some factors in this case, other than the choice of PNB or PAI, that may have contributed to this patient's varying pain scores. The patient experienced increased swelling on the right hip, and different medications were used for the PNB and PAI. While liposomal bupivacaine was FDA-approved to last 72 hours, Hussain et al. concluded that it was non-superior to plain bupivacaine [9]. Ultimately, despite the patient's increased pain and reduced range of motion in the right hip, he was still able to participate well in physical therapy and reach full recovery bilaterally after only three months.

Persistent post-surgical pain after THA can affect anywhere from 7% to 23% of patients, with significant impacts on patient satisfaction and daily life. The preexisting pain and the severity and duration of poorly controlled acute postoperative pain are identified as predictors of persistent post-surgical pain [10]. Despite the controversial roles of PAI and PNB in acute and chronic pain prevention, it is believed that aggressive and effective management of perioperative acute pain may minimize the development of spinal sensitization, thereby minimizing the transformation of acute pain to chronic pain [11].

## Conclusions

While the results for this patient favored PNB over PAI for postsurgical pain management after THA, this data cannot be generalized to all patients. It is our goal to develop a protocol for THA that targets not only acute pain but also sub-acute pain after surgery and subsequently impacts persistent postsurgical pain prevention. This will improve early discharge, patient satisfaction, and clinical outcomes.
